# Savannah Medical College

**Published:** 1857-06

**Authors:** 


### SAVANNAH MEDICAL COLLEGE.

Upon our recent trip to the American Medical Association, we had an opportunity of forming the acquaintance of two of the Professors in this Institution, and having been assured, in the most positive manner, that their recent circular was not intended as an attack upon the Atlanta Medical College, we, of course, are willing to give them the benefit of that disclaimer through our columns, and at the same time, would assure them that we are *perfectly willing*, that they should pursue their own course, and regulate the particular policy of their own institution, provided they do not attempt to bring into discredit, in an unfair way, the plan which we (in the exercise of the same right which we accord to others) have thought fit to adopt.

The truth is, we believe those controlling the Savannah College to be gentlemen, and feel rather kindly than otherwise towards the members of the Faculty with whom we have recently become acquainted, and while, from the position which they have thought fit to assume, we can not *possibly* be otherwise than antagonistic, we assure them that we are just as *friendly* as human nature can be expected to be, towards those whose declared policy strikes a blow at the Institution with which we are connected. While we have, upon a former occasion, in reference to this matter, manifested a different spirit from that which controls us at present (the present mood being the result of assurances, the sincerity of which we could not question) we must nevertheless say, that with the lights then before us, a sense of invaded rights *compelled* us to adopt the course pursued.

---

☞ Proceedings of the American Medical Association in our next number. Received through courtesy of Nashville Journal, but too late for publication.

